# Sample Preparation for Electron Probe Microanalysis—Pushing the Limits

**DOI:** 10.6028/jres.107.051

**Published:** 2002-12-01

**Authors:** Joseph D. Geller, Paul D. Engle

**Affiliations:** Geller MicroAnalytical Laboratory, Topsfield, MA 01983-1216

**Keywords:** electron probe microanalysis, grinding, mounting, polishing, quantitative analysis, sample preparation, sawing

## Abstract

There are two fundamental considerations in preparing samples for electron probe microanalysis (EPMA). The first one may seem obvious, but we often find it is overlooked. That is, the sample analyzed should be representative of the population from which it comes. The second is a direct result of the assumptions in the calculations used to convert x-ray intensity ratios, between the sample and standard, to concentrations. Samples originate from a wide range of sources. During their journey to being excited under the electron beam for the production of x rays there are many possibilities for sample alteration. Handling can contaminate samples by adding extraneous matter. In preparation, the various abrasives used in sizing the sample by sawing, grinding and polishing can embed themselves. The most accurate composition of a contaminated sample is, at best, not representative of the original sample; it is misleading. Our laboratory performs EPMA analysis on customer submitted samples and prepares over 250 different calibration standards including pure elements, compounds, alloys, glasses and minerals. This large variety of samples does not lend itself to mass production techniques, including automatic polishing. Our manual preparation techniques are designed individually for each sample. The use of automated preparation equipment does not lend itself to this environment, and is not included in this manuscript. The final step in quantitative electron probe microanalysis is the conversion of x-ray intensities ratios, known as the “*k*-ratios,” to composition (in mass fraction or atomic percent) and/or film thickness. Of the many assumptions made in the ZAF (where these letters stand for atomic number, absorption and fluorescence) corrections the localized geometry between the sample and electron beam, or takeoff angle, must be accurately known. Small angular errors can lead to significant errors in the final results. The sample preparation technique then becomes very important, and, under certain conditions, may even be the limiting factor in the analytical uncertainty budget. This paper considers preparing samples to get known geometries. It will not address the analysis of samples with irregular, unprepared surfaces or unknown geometries.

## 1. Sample Collection, Transport, and Particulate Mounting

EPMA samples originate from a large variety of sources. They may be powders, corrosion scales on a host surface, coated substrates or from bulk specimens such as metals, ceramics, glasses, and plastic or organic material. In collecting the sample one must be certain that nothing is added that will later be analyzed and mistaken for the analyte. One area of analysis where this often happens is with the analysis of small particles, which have to be collected and transported.

The challenge here is to collect the particles and embed them in a media suitable for grinding and polishing. The purpose of embedding or mounting is to hold the particle during preparation so known geometries can be established between the electron beam and x-ray detector. During analysis small airborne particles, that may range in size from sub-micrometer and up, are often filtered or collected electrostatically. Particles may also be suspended in liquid form. How can such small particles be collected and transported for analysis? Air and liquid filtration may embed these particles into the depth of three-dimensional filters, preferably single surface filters can be used with the particles populating on a single surface (polycarbonate materials preferred due to its chemical inertness), but with much reduced conductance resulting in far less volume filtered. With depth filtering the filter can be dissolved concentrating the particles for later surface filtration or embedding. There is a risk, however, that the solvent used will react with the analyte by either putting it into solution, corroding the sample, or causing a chemical reaction resulting in a new compound or that impurities in the filter itself be mistaken for the analyte. Larger particles may exist as scale and be simply scraped off a surface taking care not to sacrifice the scraping tool in the process. For instance, this author has had the unfortunate experience of finding tungsten in unknown particles. With no tungsten used in the process that generated these particles the origin was later determined to be a tungsten probe that was used to dislodge the particle. Unknowingly, a result was produced that had nothing to do with the sample.

Particles may be suspended in oil that were created while an engine or mechanical device was in operation. Concentrating the particles can be done by removing them from the liquid with a tweezers or fine probe while being observed under a microscope. They can also be filtered, as described above, except care must be taken to use solvents that are compatible with the filters and the particles. Sometimes particles are collected with adhesive tape. While this may be easy for collection, it can be difficult to completely and cleanly remove them. Adhesive residue on the particles may be very difficult to remove, even with solvents and sonication. If the particles are embedded with adhesive residue they may move during grinding and polishing. This results in poor surface finish or loss of the particle. We prefer using the Post-It™ note type of adhesive paper. While particle bonding may not be as good the adhesive is thinner and remains on the paper providing less chance for contamination.

**Fig. 1:**
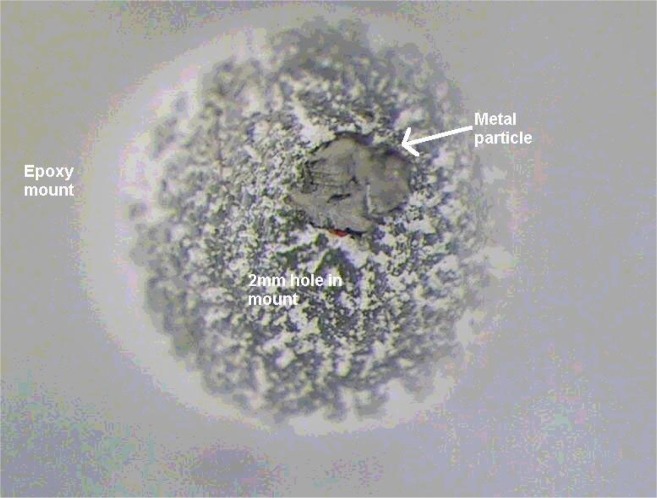
Epoxy mount prepared for sectioning micrometer sized particles. A metal sample (10 µm) is at the hole bottom.

Once the particles are collected they are next prepared for polishing, the purpose of which is to establish the necessary take-off angle for analysis. Clear embedment media offers the technician the advantage of being able to observe the particles during the preparation process. We have had much success using two-part epoxies (available from the major metalographic suppliers) that cure overnight. Epoxies generally have very good adhesion to the samples. In general, slower curing epoxies are harder and have better edge retention. Other embedment media, such as cold mounts and acrylics that cure very quickly may not bind well to particles, have poor edge retention and decompose rapidly from electron beam irradiation. Clear hot mount materials that are hardened under high pressure and temperature may cause the particles to be redistributed over too large a volume with the end result being only a few particles exposed over the polished surface at the same time. A technique which we use, and may have been used by others, employs a cured, vacuum degassed, epoxy mount. Several small (~2 mm) holes are drilled to the same depth in the mount. The bottom of the holes has a “V” shape from the drill point. The particles are placed, using a probe, into each hole making sure they find their way to the bottom (Fig. 1). The holes are then filled with freshly mixed epoxy, vacuum degassed, and then examined under the stereo microscope to insure the particles are still at the hole bottoms. Some of the holes at the periphery of the mount can be filled with a marker material, such as small ball bearings or wires. This will enable the technician to rapidly locate the plane of the embedded particles. Since there will be entrapped gas bubbles in the uncured epoxy the mount should be carefully vacuum degassed, making sure not to volatilize the hardener in the process. If this happens the epoxy will not harden.

The mount is then ground with successively finer grit abrasives while intermittently observing the depth of the markers below the mount surface. When the particles become exposed, or nearly exposed, the final polishing steps are initiated. This technique also works well for analyzing wires and glass cylinders, such as fiber optics.

## 2. Preparing Samples or Calibration Standards That are Available Only in Particulate Form

**Fig. 2:**
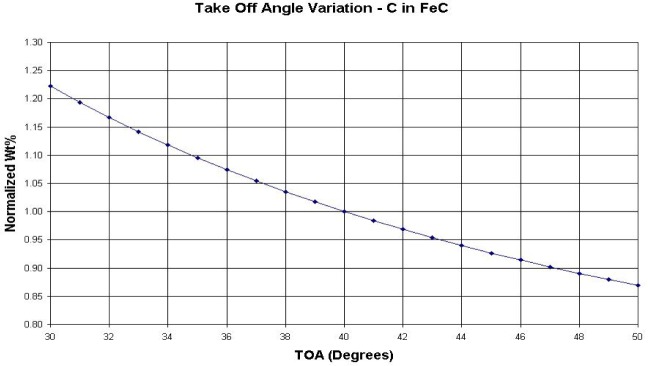
Take off angle variation for carbon in iron where a 1° error in take off angle at 40° results in a 2 % relative error for carbon.

1. The choice of calibration standards can be equally as important as the preparation to reduce the analytical uncertainty. The ZAF calculation is used in a two-step process. The first is to convert the intensities of the compound standard used for x-ray intensity calibration into *k*-ratios, where the *k*-ratio is the counting rate ratio between the unknown and standard. After the sample data is collected those x-ray intensities are converted to *k*-ratios then run through the ZAF calculation program for determining concentrations. There are many variables in the ZAF calculation that affect the outcome. The selection of mass absorption coefficients, stopping power, fluorescence corrections, and errors in the take off angle result in variations of several percent. However, if the standard chosen is the same composition as the unknown no corrections need to be made. If an exact match is not available the analyst should choose a standard whose elements were in the same type of matrix and concentration to minimize the corrections. This was the basis for the early Bence-Albee [1] program for mineral analysis—and it worked quite well. The difficulty in using this technique is the lack of reliable standards. Well characterized mineral standards, in particular, are in collections in museums such as the Smithsonian Institution in Washington, DC and many universities that have EPMA laboratories. Since they are inshort supply they are difficult to acquire, often available only on a barter basis. Even so, there are many naturally occurring standards that are simply unavailable. If the sample is not flat causing unknown errors in the take off angle the proper corrections to the data will not be made. For instance, for 1 % carbon in steel (Fig. 2) a 1° error in take off angle at 40° results in a 2 % relative error for carbon.

## 3. A System of Calibration Standards

We have developed a system of standards (UHV-EL) that uses a 25 mm diameter stainless steel holder with up to 37 holes, each being 3 mm in diameter. In this system each standard is individually prepared, with no materials other than the embedding medium, to prevent cross contamination that occurs when preparing materials of differing hardness. The holder is designed so that the polished surface of each standard when placed in the holder is top referenced. This automatically aligns the standard with the plane of the holder and provides for a predictable take off angle and a constant working distance when moving between standards. The holder is laser engraved uniquely identifying the position of each standard. These engraved markings are imaged either optically or with the electron image. The 250, or so, standard materials available can be selected from pure elements, compounds, glasses, alloys and minerals. Each standard is polished using a procedure developed specifically for that material, rather than polishing a surface containing a large range of materials with different hardness. This also prevents cross contamination where materials of different hardness contaminate each other. The standards are held in place with a stainless steel clip and are easily removable. General preparation guidelines will be given later in this manuscript.

We try to select standards that can be directly fabricated into 3 mm disks. The easiest to work with are foils that can be punched. Ceramic materials and glasses can be cored with mechanical or ultrasonic drill. However, many materials are only available in powder form. Unfortunately, chemical suppliers often engineer their processes to produce the minimum size powder grains. A small number of these materials, all compounds, are sieved, and while they pass through 300 mesh screens and often are agglomerated and have individual sizes approaching 1 µm. This is too small for microanalysis considering the electron scattering volume. However, other sources may not be available. A few examples are WO_3_ and MoO_3_, for which the correction factors, including the mass absorption coefficient, is not well known. These powder grains must be mounted for grinding and polishing.

**Fig. 3:**
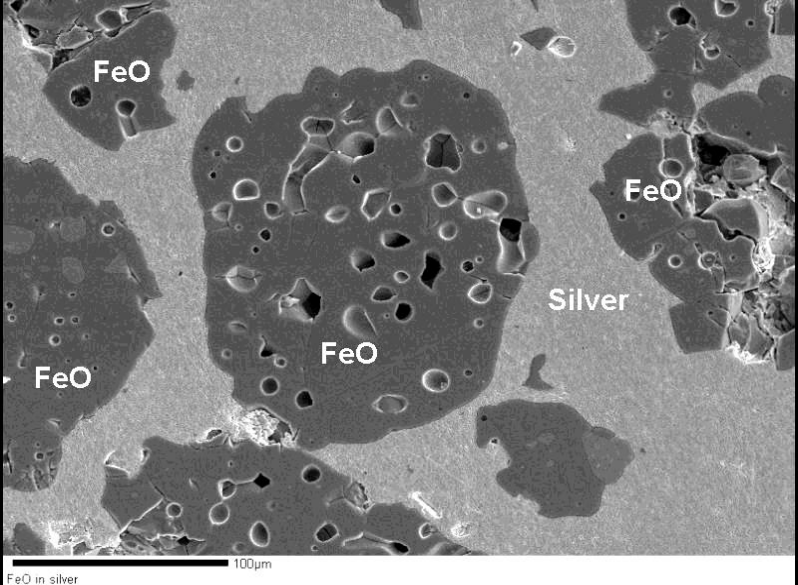
FeO calibration (powder grains) standard mounted in silver and flat polished.

We have selected silver flake (or tin flake for preparing sulfides since sulfides reacts with silver) as a mounting medium since they have good electrical conductivity, bleed the charge and help to conduct away the heat generated from the electron beam sample interaction and do not add an excessive number of peaks to the x-ray spectrum at analysis time. These metals are mixed with the analyte, pressed at ≈1.4×10^3^ GPa pressure and mildly annealed to densify and help keep the disc together for further processing. The composite disc is then ground and polished, revealing the polished analyte surfaces. As an example the SEM photograph in Fig. 3 shows iron oxide grains in silver.

## 4. Sizing the Sample

In many cases samples need to be reduced in size to fit into the analytical position in the EPMA chamber, into a metallurgical mount, or a polished section. The sizing method used, if too aggressive, can cause deep damage to both morphology and chemistry which may not be removed during preparation. Sizing methods used involve cleaving, scribing fractioning scissors, sawing, cutting, torching, and wire EDM (electro discharge machining). In all cases it is imperative to be aware of the damage depth caused. Subsequent operations must completely remove the damaged material.

For semiconductor wafers cleaving is accomplished by placing a sharp pointed object on one side of the wafer and applying pressure at the opposite side starting the cleave. If the cleave direction is oriented with the crystallographic plane a straight cleave will follow. Unfortunately the cleaved edge will rarely be at right angles to the wafer resulting in an unknown geometry. For EPMA instruments, with an optical microscope having a shallow depth of field, it may be possible to find a location with the proper geometry for analysis.

Scribing thin, brittle samples is accomplished by using carbide or diamond pointed tools to define a fracture plane. The revealed surface will usually require polishing unless suitable areas for analysis can be located.

Metal foils and soft coated materials can be cut with scissors or shears. Care must be taken to removed material smeared across the cut surface.

Sawing operations require careful consideration of damage depth and possible sample contamination when using abrasive coated wheels. Over-aggressive sawing can cause deep sample damage, generating cracks and heat affected zones beyond the depth for which grinding operations can remove them. Metallographic cut-off saws using ceramic blades may generate heat burning the sample surface causing melting, oxidation or the removal of surface coatings. Liquid cooling during the cut is recommended. Low speed diamond saws cut slowly, but leave a smooth surface and cause very low sample deformation. To prevent contamination from the cooling liquid used during cutting the sample can be dry cut. Rotation of the sample during cutting^1^ helps to increase the cutting speed (up to ten times), polishes the surface, and allows for a larger area to be cut. The choice of blade used is critical.

Metal samples can be cut with shears or pliers. Sample deformation again is a concern. Using a welding torch to cut a sample brings concerns about heat modification of the sample. Finally, the wire EDM (electro discharge machine) is a very useful way to cut electrically conductive samples. The wire EDM cuts by sparking its way through the sample, which is immersed in high resistivity water. It is very useful for cutting small samples from large pieces. The deformation zone is rather shallow so it is easy to grind through the damage. The down side to this technique is the high cost of the equipment and operator skill required, since wire EDMs are generally computer controlled.

## 5. Mounting the Sized Sample

Samples large enough to be held by hand or another mechanical device during grinding and polishing may not need to be mounted. There are several choices to be made for mounting materials. Some of these are thermoplastic, acrylic cold mount materials, epoxies, and bakelite, diallyl phthalate or methyl methacrylate hot mount materials.

Sample orientation in the mount is often important. Many times multiple small sized samples are placed in the same mount for simultaneous polishing. They can be held in place in a number of ways. The metallurgical supply houses sell metal and plastic clips. Samples can also be placed in sparing amounts of glue or epoxy and allowed to set before final mounting. They can also be placed on double sided tape. All the mounting materials below, except the hot mount materials, do not move the samples excessively. The mounting pressure with hot mount materials may cause differential movement changing the orientation.

Special consideration should be given to the mounting technique if EPMA analysis is done at the samples edge, to prevent contamination from the electron beam degrading the mount. One application is in the measurement of low concentration (<1 %) carbon in steels to determine case hardening depths. Contamination from decomposed acrylic or epoxy will increase the carbon x-ray intensity giving false high readings. The mounting media formed at higher temperatures (carbon or metal filled hot mounts) have the best characteristics since no additional conductive coating is necessary.

Thermoplastic materials, like Crystalbond^2^ 509™ have low melting temperature (≈80 °C) and are soluble in acetone. This material should not be placed in a vacuum system due to outgassing and degradation if struck by an electron beam. 509 is very useful for holding samples in position for low speed sawing and polishing. It is also useful for final polishing and then removing the mount materials so that the polished surface can be viewed along with the adjacent non-polished area (which could be a fracture, crack or surface contamination or scale).

Acrylic cold mount materials set very quickly but do not bond well to surfaces and have low hardness. Some acrylics rise in temperature by about 55 °C during curing. Poor bonding leaves gaps that allow abrasives and slurry to collect and come out during later preparation steps where finer abrasives are used. This causes scratches and outgassing when placed in vacuum. The outgassed material can contaminate the sample surface. Acrylic materials offer low resistance to electron beam exposure, decomposition upon contact and contamination of the sample. They are also too viscous to fill low density samples.

Two part epoxies better than acrylics for mounting. They bond well to surfaces, have higher hardness and are of sufficiently low viscosity to fill low density sample voids. They can also be filled with hard material, such as alumina ceramic, matching the hardness of the sample to help maintain edge retention. They have longer set times than acrylics, up to 24 hours. During curing the exothermic reaction may raise the temperature by about 80 °C, depending upon the amount of hardener used. More hardener and/or heating the mount in an oven decreases the cure time. Epoxies decompose under electron beam irradiation. Epoxy mixes have a lot of air dissolved. The air bubbles in the cured mount may retain abrasives that can scratch the specimens later when finer polishing grits are used. A freshly mixed epoxy can be vacuum degassed, taking care not excessively volatize the hardener, changing the mix and interfering with proper curing. Vacuum mounting also helps the epoxy flow into porous surfaces much better. To ensure edge retention samples can be electroless nickel-coated or coated with epoxies filled with ceramics, such as Varian^3^ Torr-Seal™.

Bakelite hot mount materials require high temperature (up to 150 °C) and pressure. They form hard mounts and offer good stability from electron beam irradiation. With the addition of carbon or metals like copper some are electrically conductive. Unfortunately, they are too viscous to fill voids in porous samples. One must be careful how the sample is placed in the mount, if the sample is too large or too close to an edge the mount may crack due to differential expansion causing-gaps between the sample and mount. Seepage of polishing oils in bonding gaps during polishing results in sample scratching. Seepage can also occur when the sample is placed in a vacuum that results in contamination of the sample, and possibly, the EPMA. Unfortunately, these high viscosity mounting materials do not fill porous samples as well as epoxy. Analysis of porous materials, like ceramic catalysts, is an important EPMA application. To prevent cracking the manufacturer’s cooling recommendations must be carefully followed after the hot mount is formed.

Some hot mount materials are filled with carbon or copper to make them conductive. These materials are supplied as pellets or spheres that liquefy at process temperature and pressure. We find it helpful to grind the materials into a fine powder before use—this minimizes movement and helps the material flow around the sample.

**Fig. 4:**
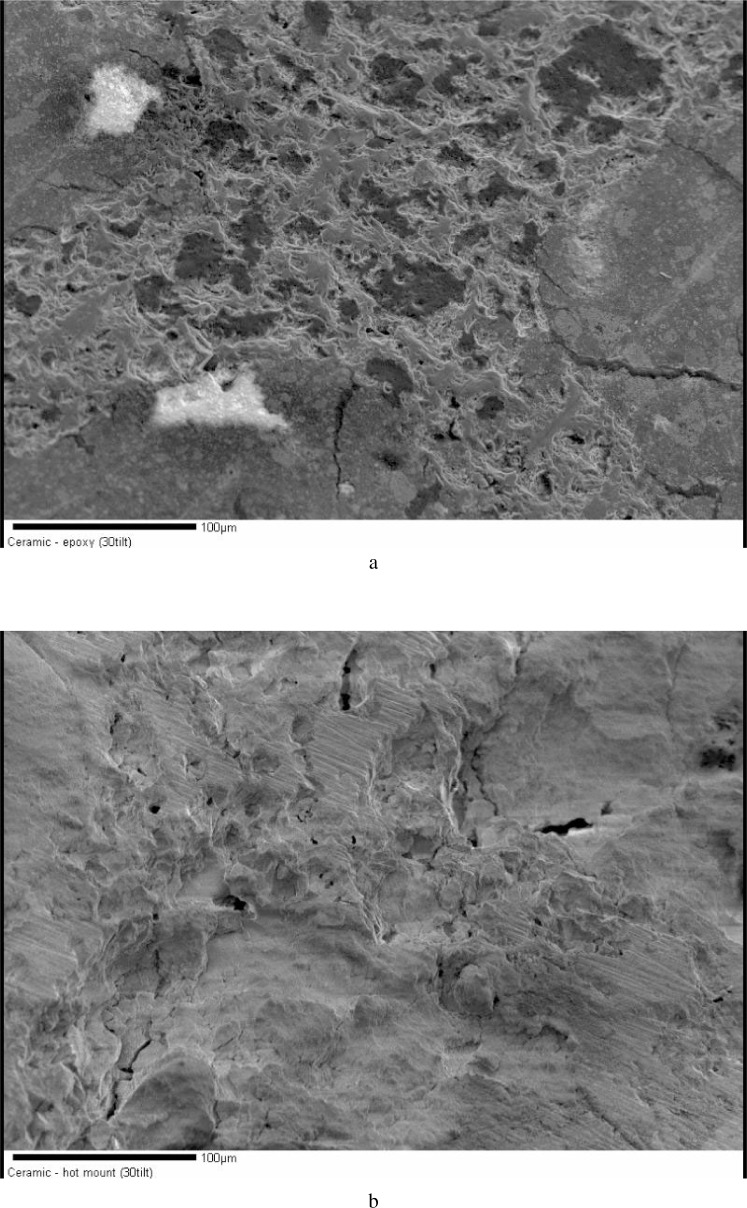
a. Porous ceramic honeycomb material vacuum embedded in epoxy and polished. Some areas are flat for analysis. b. Same material hot mounted. No flat surfaces are seen.

**Fig. 5:**
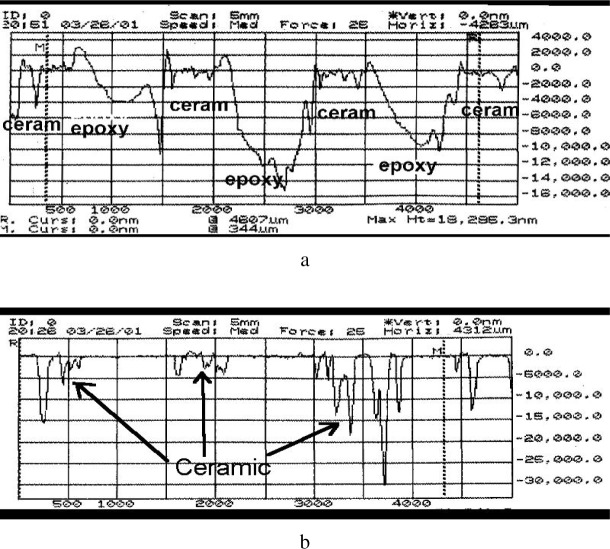
a. Same sample as in Fig. 4. The softer epoxy areas are preferentially polished while the epoxy filled ceramic areas are relatively flat. b. The hot mounted sample surface is flush with the void, but the porous ceramic areas are very rough.

A comparison of two different preparations (hot mount and epoxy) of a porous, loosely bound, honeycomb like ceramic sample is shown in Fig. 4a and 4b. In the hot mount preparation (Fig. 4b) no flat surface is observed since the mounting material did not fill the voids, while the epoxy preparation binds the sample for polishing (Fig. 4a), and some flat surfaces are observed. The darker areas are epoxy. Figures 5a and 5b show a profilometer scan across the mount for both preparations. The upper figure is from the epoxy mounted sample. The large voids were epoxy filled. Epoxy is much softer than the ceramic and is preferentially removed during preparation. The ceramic is much flatter than that for the hot-mount preparation. Since the hot mount material did not hold the ceramic in place during polishing much material was lost in preparation.

## 6. Grinding

The next step in getting a flat polished surface is to grind the bare or mounted material. There is a range of media available including alumina, silicon carbide, and diamond abrasives. These materials are available for use as loose powders or fixed onto a substrate—like paper or metal, in the case of diamond. In our laboratory we prepare a large number of materials with different hardness and are always concerned about cross contamination. For that reason we prefer to use low cost, disposable silicon carbide paper. While diamond discs may offer some advantages for production work on the same materials day after day, we cannot afford to contaminate our standards or customer supplied materials.

The grinding technique is very important. The surface area being ground, and the loading weight should be varied to optimum conditions for each material.

The grit size is also important. We find that 240, 400, followed by 600 silicon carbide grit is satisfactory for most samples. The basic idea behind grinding is that each step should remove the damage from the previous grit used. Embedding grit is a big problem, especially with soft materials. There is very little published literature on this subject. Factors that affect embedding for a given material are; the type and size of abrasive used, and the force applied during grinding and polishing and the affinity between the grit and the sample.

**Fig. 6:**
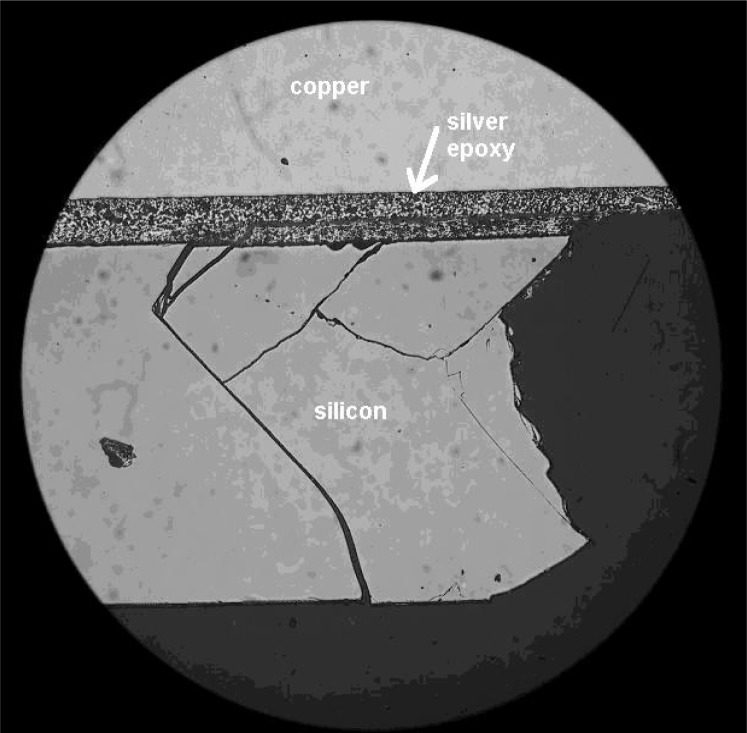
Epoxy mount copper sample with silver epoxy and copper layers. Cracking in the silicon is from over aggressive grind with a high speed rotary tool.

**Fig. 7:**
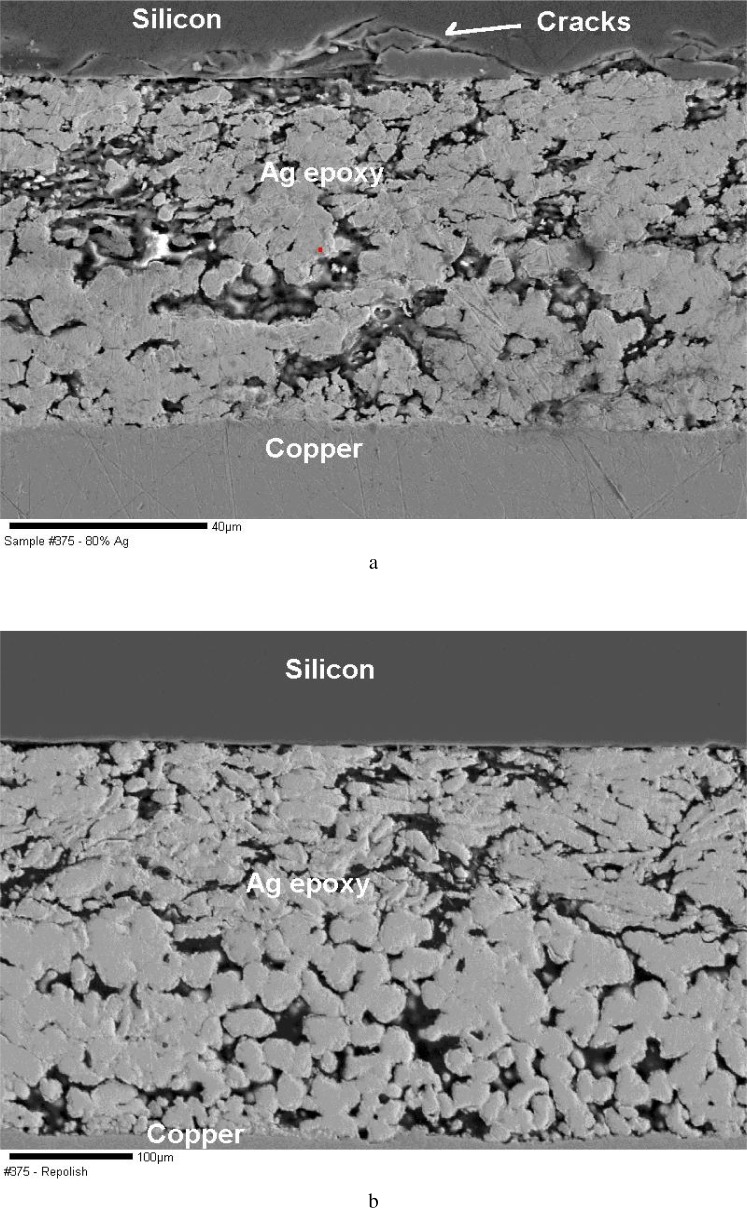
a. Same sample in Fig. 6 after final polishing. Fine cracks are seen at the silver/silicon interface. b. After properly removing the damaged layer the interface is free of cracks. 8. The flat polished flat surface in the sample from Fig. 2 was transformed into a pearlite structure after etching with HNO_3_. How can one determine the take off angle?

The example in Fig. 6 shows a silicon substrate that is coated with silver epoxy and then copper. The cracks seen in the silicon are from cutting with a high speed rotary grinder. At higher magnification, the SEM photos in Fig. 7a shows fine damage at the silver/silicon interface after final polishing, while Fig. 7b show the interface after further grinding and polishing past the damage layer.

## 7. Polishing

Polishing is the final step in sample preparation, although chemical or electro chemical etching may be used (more about that later). Vander Voort’s metallography book is an excellent reference [2] on all phases of metallograpy. The ASM Metals Handbook [3] also is a good source and has a lot of information on the preparation of various materials.

Abrasives used for polishing are generally finer than those used for grinding, although the choice of materials is similar. We prefer not to use CrO_2_, SiO_2_, CeO_2_, or Al_2_O_3_ since they easily embed themselves into the sample. In addition, many of the samples we analyze contain Cr and Al. Diamond in a water soluble paste, for easy washing, is our choice in the sequence of 6 µm, 3 µm, and 1 µm grit sizes. Embedded diamond in a sample is relatively easy to recognize and does not interfere with most analytical jobs. For those samples that are water soluble, such a NaCl and KI we polish with kerosene as a lubricant for the diamond paste in a water and oil soluble carrier. Clean up is then done using methanol. Another common final polish material used in the industry is sub-micrometer silica gel. This produces a high luster on the sample surface, but suffers from the possibility that this fine abrasive will embed itself in the sample.

The choice of polishing cloth is critical for some samples. To keep samples flat the harder cloths with short naps are generally used. For softer samples a long nap cloth with light sample pressure is preferable. Some general recommendations for different types of materials are given below:

### 7.1 Soft Metals

We have not found a mechanical way to polish indium, not that we haven’t seen a shiny scratch free surface (which was full of 0.1 µm diamond). Indium can be cut with a glass or diamond microtome knife. Other soft metals like Pb, Au, Pt, Ag, and Pd are polished under the following guidelines.

1. Remove as little material as possible. Start with a flat surface.
2. Load the silicon carbide paper with bees wax. This help keeps the SiC from embedding in the metal.
3. Try to start polishing with 3 µm diamond without using silicon carbide. Use light pressure for a long time reverting to SiC when necessary.
4. Using a high nap cloth helps keeping the diamond out of the metal.

### 7.2 Hard Metals

For metals such as Ti, V, Nb, and Zr we have found it is better to polish with 3 µm diamond and avoid using 1 µm, going directly to 0.1 µm for a short time. The 1 µm diamond leaves scratches that are difficult to remove.

1. Aluminum and copper: these metals scratch easily, requiring 0.1 µm to finish.
2. Fluorides: water can leach out the fluorine, changing the stoichiometry.
3. Combined hard and soft materials: for instance, gold coatings on ceramic. In general, light pressure is used for long periods, with low nap cloth to help keep the sample flat. For refurbishing standards that are embedded and flat polished in one mount (this is not the way the UHV-EL standards are made) one must be careful about water-soluble standards.

## 8. Etching

**Fig. 8:**
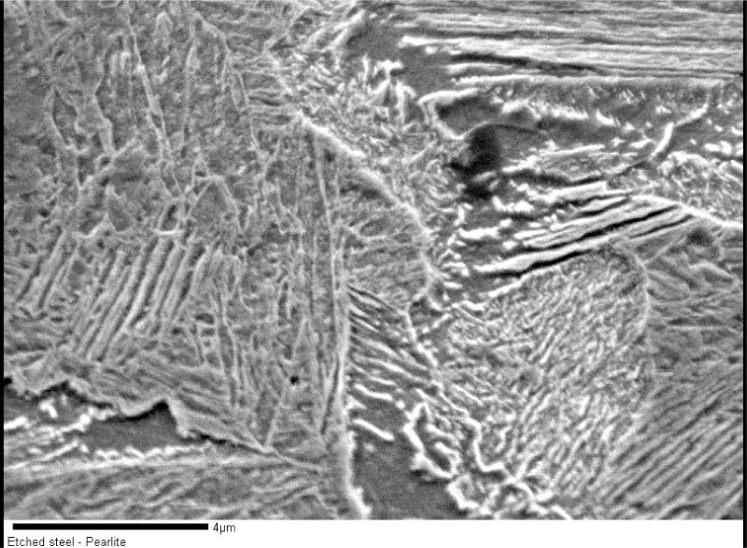
The flat polished flat surface in the sample from Fig. 2 was transformed into a perlite structure after etching with HNO_3_. How can one determine the take off angle?

Etching is often used to reveal the samples grain structure. The etching mechanism is selective removal of the sample by either chemical means (the etchant dissolving different phases in the sample at different rates), by oxidation or reduction, or by removal of the sample at different rates according to the crystallographic orientation. In any case the surface flatness is compromised, as is the near surface density. While microstructure can be readily seen, the totals of the EPMA analysis are usually low. The 1 % carbon in steel sample shown in Fig. 2 was chemically etched with HNO3. The SEM photo in Fig. 8 shows that the flat polished flat surface was transformed into a pearlite structure. A profilometer scan revealed that the height variations are on the order of 1 µm, while the structural variations are much finer. Without even considering chemical changes from etching one can visualize what a rough surface the electron beam sees. The take off angle on this surface is then indeterminate. An EPMA analysis using a 1 µm beam is unlikely to be orthogonal to the surface.

## 9. Conclusion

Sample preparation for EPMA is critical to good analytical results. Like the proverbial “weakest link in the chain” no data correction routine will fix errors made at the start of the process, which include collection and transport of the sample to the analytical laboratory. Once the sample is in the laboratory for preparation, two areas that we have discussed are contamination of the sample with foreign material (that can be either from sample handling prior to preparation or the embedding of polishing abrasives during the preparation process) and establishing known geometries (which are critical to the correction algorithms) between the sample surface and electron beam. This encompasses mounting, grinding and polishing the specimen to achieve a flat surface.

## Footnotes

1 Counter-Rota-Cutter, patent *#*4,949,605 from Geller MicroAnalytical Lab, Topsfield, MA 01983

2 Crystalbond 509 is manufactured by Armeco, Ossining, NY.

3 Torr-Seal is manufactured by Varian Associates, Lexington, MA.
